# Hazards of steroid injection: Suppurative extensor tendon rupture

**DOI:** 10.4103/0970-0358.63971

**Published:** 2010

**Authors:** Colin Yi-Loong Woon, Ee-San Phoon, Jonathan Yi-Liang Lee, Siew-Weng Ng, Lam-Chuan Teoh

**Affiliations:** Department of Hand Surgery, Singapore General Hospital, Singapore; 1 Department of Hand and Reconstructive Microsurgery, National University Hospital, Singapore

**Keywords:** Tenosynovitis, tendon rupture, extensor tendon, infection

## Abstract

Local steroid injections are often administered in the office setting for treatment of trigger finger, carpal tunnel syndrome, de Quervain's tenosynovitis, and basal joint arthritis. If attention is paid to sterile technique, infectious complications are rare. We present a case of suppurative extensor tenosynovitis arising after local steroid injection for vague symptoms of dorsal hand and wrist pain. The progression of signs and symptoms following injection suggests a natural history involving bacterial superinfection leading to tendon rupture. We discuss the pitfalls of local steroid injection and the appropriate management of infectious extensor tenosynovitis arising in such situations.

## INTRODUCTION

Local injections of corticosteroid to the hand and wrist are not without risk. Trigger finger, de Quervain's tenosynovitis, carpal tunnel syndrome and basal joint arthritis are some conditions for which steroid injection is accepted treatment. Although such injections are often done in the outpatient setting with minimal risk and immediate return to activity, the physician must remember that introduction of skin flora into the tendon sheath can result in suppurative tenosynovitis with disastrous complications.[1]

Infectious flexor tenosynovitis is better characterized[2] in the literature than extensor tenosynovitis, with the latter more often associated with rheumatoid arthritis,[3] systemic lupus erythematosus, gout and pseudogout. We report a rare case of rapidly progressive suppurative extensor tenosynovitis with tendon rupture following improper administration of local steroids and review the caveats of this procedure.

## CASE REPORT

A 55-year old right-handed male with Type 2 diabetes mellitus on oral hypoglycemic agents presented initially with pain over the right hand dorsum. At the clinic, after cleansing with alcohol swabs, two subcutaneous injections of 2ml triamcinolone acetate (10 mg/ml) and 2 ml lignocaine (1%) were administered to the dorsum of the 2^nd^ & 3^rd^ metacarpophalangeal joints (MCPJ) and 4^th^ & 5^th^ MCPJs (extensor zone V). He returned with persistent pain over the wrist ten days later and was given a similar subcutaneous injection in the region of the dorsal wrist joint (zone VII). Two months later, he developed painful dorsal hand swelling that did not respond to oral antibiotics. The swelling then spread to the mid-forearm at which time he was referred to our institution.

At the emergency room, he demonstrated tender dorsal forearm swelling up to the ulnar four MCPJs. Active range of motion (ROM) at the ulnar four MCPJs was 0 to 90°. Neurological examination was normal. Radiographs were remarkable only for soft tissue swelling. Citing personal reasons, he remained adamant against hospital admission for intravenous antibiotics and surgical drainage under general anesthesia. Instead, less thorough surgical debridement under Bier's block in the emergency room was performed. Utilizing two incisions over the regions of maximum fluctuance, frank pus was expressed from the subcutaneous tissue and between the tendons. Tenosynovectomy was limited by ischemic tourniquet pain. Although surrounded by purulent material, extensor tendons were visualized and found to be intact and continuous. The wound was dressed and he was discharged with oral broad-spectrum antibiotics and an early follow-up appointment.

**Figure 1:**
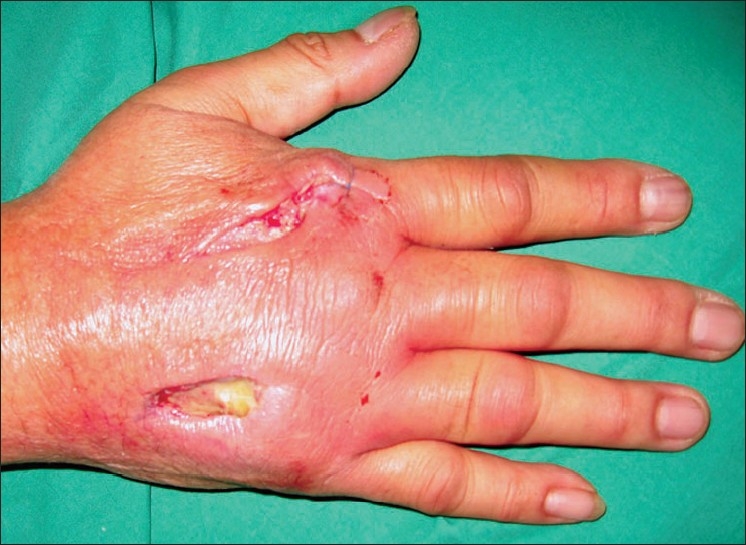
Wound appearance two days after initial debridement

At scheduled review two days later, there was residual soft-tissue erythema and induration, and small amounts of purulent discharge at the incision sites [Figure 1]. He also complained of one-day history of loss of active MCPJ extension in right index, middle and ring fingers. Examination confirmed dropped fingers. Neurological examination was unremarkable. Active range of MCPJ motion was 30 to 90° but passive MCPJ extension was normal. On laboratory investigations, white cell count was 11.7 × 10^9^/L, erythrocyte sedimentation rate was 40mm/h and C-reactive protein was 123 mg/L. Rheumatologic tests were normal.

**Figure 2:**
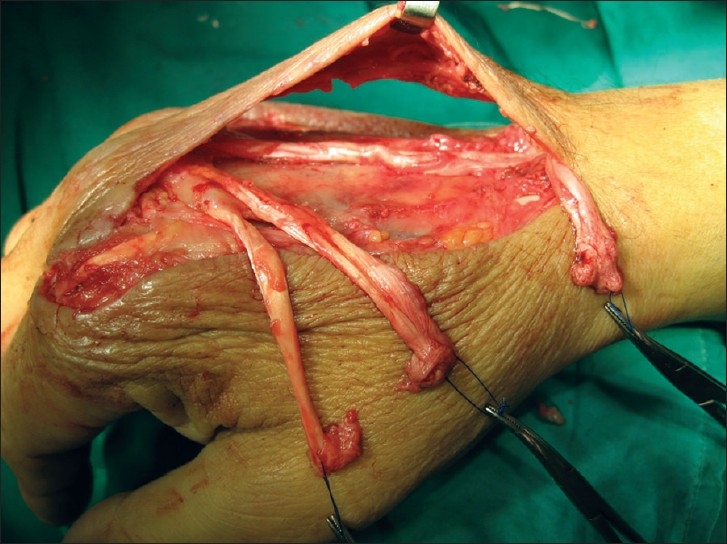
Intraoperative findings at repeat debridement showing multiple ruptured extensor tendons. Tendon ends were frayed and necrotic

This time, he consented to hospital admission. Exploration under general anesthesia and tourniquet ischemia revealed multiple ruptured tendons. The extensor indicis proprius (EIP) was ruptured in zone VII, with proximal retraction under the extensor retinaculum. The extensor digitorum communis (EDC) tendons to the index finger, middle finger and ring finger were ruptured in zone V, zone VI and zone V, respectively. All tendon stumps were necrotic and frayed [Figure 2]. Extensive debridement and pulsed lavage irrigation was performed. Tendon ends were trimmed back and protected under the skin bridge for later repair. Perioperative amoxicillin/clavulanate was switched to cloxacillin for intraoperative cultures positive for methicillin-sensitive *Staphylococcus aureus* infection.

**Figure 3:**
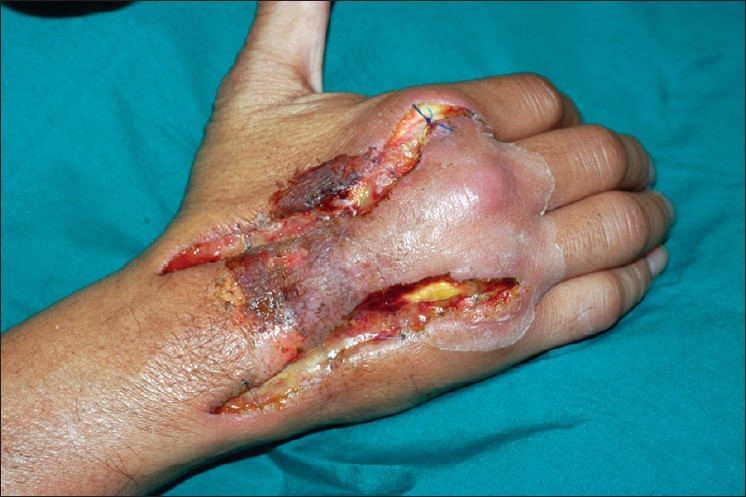
Intraoperative wound appearance prior to tendon reconstruction. Superficial skin necrosis is seen to involve the proximal half of the bipedicled skin bridge

**Figure 4:**
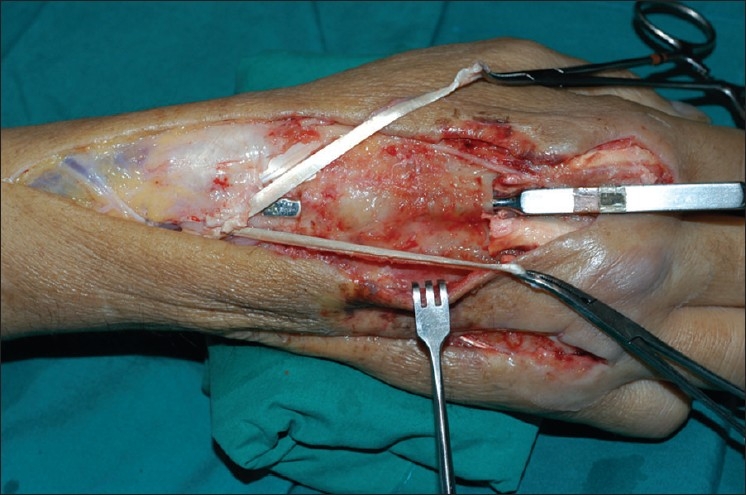
Extensor tendon reconstruction with folded-over PL graft. Proximal attachment to common extensor bundle of index, middle and ring finger. Distal attachment to EIP and EDC of index finger (radial limb) and EDC of middle and ring finger (ulnar limb)

**Figure 5:**
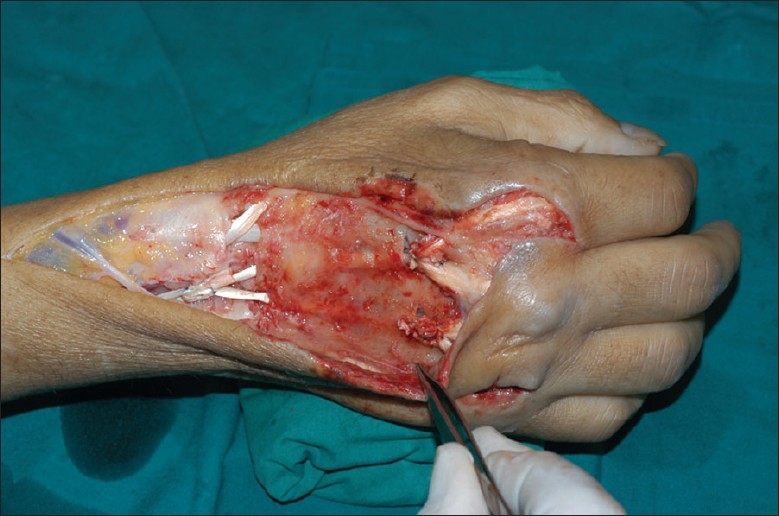
Folded-over palmaris longus graft tunneled deep to extensor retinaculum

One week later, infection resolved [Figure 3] and he underwent reconstruction with a free doubled-over palmaris longus (PL) bridge graft. The proximal extensor tendons to right index, middle and ring fingers were trimmed back to zone VII. The PL graft was doubled over and weaved into the common bundle of extensor tendons proximally. The two distal ends found attachment to the distal ends of right EIP and EDC to the index finger (radial limb), and EDC to the middle and ring fingers (ulnar limb) [Figure 4]. The PL graft was tunneled under the extensor retinaculum [Figure 5] to buffer the repair and enhance gliding. A patch of ischemic skin over the proximal skin bridge was de-epithelialized and found to have healthy dermis. The bipedicled skin bridge was pulled radially, over the main PL graft body to close the radial defect, while the ulnar defect was covered with split-thickness skin graft. Operative tissue cultures were negative.

**Figure 6:**
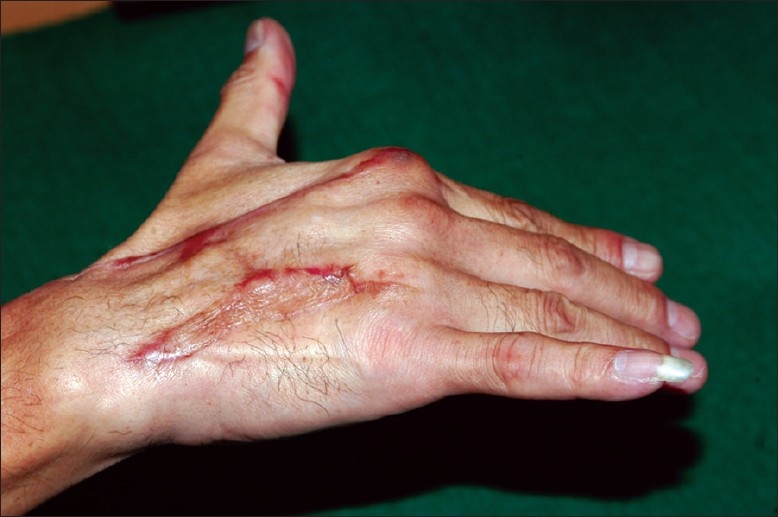
Appearance at 6 months after tendon reconstruction

He was started on active hand therapy at one month. At 6 months, all wounds had healed and there was no evidence of recurrent infection [Figure 6]. At 1 year, there was residual extension lag at the 2^nd^ to 4^th^ MCPJs (active ROM, 25 to 90°) but he was satisfied with the overall outcome.

## DISCUSSION

This case illustrates how inadequately treated infectious extensor tenosynovitis can rapidly lead to tendon rupture.[1] In addition, a few notable points arise: (1) While steroid injections are accepted treatment for many conditions, they are not innocuous and should be administered only when clinically indicated. Administration without thorough evaluation of vague symptoms of pain should not be performed. (2) Sterile technique is important. Inattention to sterile technique leads to inoculation of skin flora into the tendon sheath.[4] S. *aureus* inoculation produces early cellulitis that rapidly evolves into a subcutaneous abscess, and allows bacteria to penetrate the subaponeurotic space between tendons and invade the tendon sheaths,[5] leading to characteristic findings noted at initial surgery. Sterile technique must be emphasized for diabetics[4] and drug addicts. The former are at greater risk because of impaired cellular immunity, small vessel angiopathy, and delayed presentation associated with peripheral neuropathy, while the latter are compromised because of tendon sheath injection of foreign material. (3) Patients must be advised that pain exacerbation following injection, together with swelling, erythema and local warmth, indicates bacterial superinfection and necessitates further evaluation. (4) There is no place for limited debridement in the management of these infections.

### Tendon rupture

Tendon rupture occurred in a stepladder fashion at zones V through VII, coinciding with the sites of local injection suggesting a predominantly infectious aetiology. As the extensor tendons lack annular and cruciform pulleys, bacteria spread proximally forming multiple foci distinct from the point of entry,[1,4] producing a staggered rupture pattern.

Tendon rupture usually occurs as a late consequence of bacterial infection. Although he presented with signs of localized sepsis, this patient's refusal for acute hospitalization and formal debridement may have added unnecessary delay and allowed progression to tendon rupture.

In addition, both needle introduction and steroids may have played a secondary role. (1) Introducing a bevelled needle tip results in mechanical fibre separation and tenocyte death, reducing tensile strength by up to 60%,[6] a risk that is increased with multiple passes. It is thus advisable to withdraw the needle slightly if resistance is encountered at the start of the injection.[7] (2) Local steroids have contributed to rupture of flexor and extensor[1] tendons, Achilles tendons and patellar tendons. Steroids produce collagen degeneration,[7] inhibit tendon repair, delay tendon-sheath healing, and lessen the breaking point of sutured tendon. The effect of steroids is cumulative, with rupture occurring anywhere from 6 weeks to 4 years later.[6] In one report, 29 injections into each carpal tunnel over seven years resulted in flexor tendon rupture of bilateral ring and little fingers.[8]

### Pillars of management

The pillars of management include empirical broad-spectrum intravenous antibiotics, prompt thorough surgical debridement and delayed closure.[5] Excisional debridement must be prompt, aggressive and repeated as necessary where delay in resolution occurs.

The first temporizing operation was limited by tourniquet pain. Various authors have established upper limb tourniquet tolerance to be approximately 20 minutes in conscious volunteers.[9] Under ideal circumstances, general anesthesia should have been used to allow thorough exploration without tourniquet discomfort. Where there is swelling and induration, unhurried exploration is necessary to distinguish suppurative tenosynovitis from uncomplicated subcutaneous abscess. Thorough tenosynovectomy of involved tissue is necessary should the former be present. The approach should be through a single dorsal midline or S-shaped incision as placing more incisions in an infected field only increases the risk of skin bridge necrosis.

In conclusion, steroid injections are not innocuous. The possibility of infection and tendon rupture must be conveyed to the patient. Repeated steroid administration increases the risk of bacterial inoculation and tendon rupture.[8] In cases presenting acutely with suppurative extensor tenosynovitis, early aggressive surgical debridement is critical.
